# Conservative Therapy in Two Cases of Non-Hodgkin Lymphoma of the Penis: Case Reports With Review of the Literature

**DOI:** 10.4021/wjon438w

**Published:** 2012-02-19

**Authors:** Giampaolo Delicato, Giulio Baffigo, Daniele Bianchi, Giuseppe Farullo, Stefano Signore, Edoardo Tartaglia, Francesco Corvese, Vincenzo Ferdinandi

**Affiliations:** a Deparment of Urology-Santo Eugenio Hospital Rome, Largo Umanesimo, 10 - 00144 Rome, Italy

**Keywords:** Lymphoma of the penis, Genital localization of lymphoma, Chemotherapy

## Abstract

The malignant lymphomas rarely occur in the genito-urinary tract and particularly penis lymphomas are extremely uncommon. Frequently they do not have any specific symptoms and the diagnosis is delayed even in presence of a penis node. In our hospital we observed two patients affected by Non-Hodgkin Lymphoma (NHL), one of them with a primitive disease. Both cases were sexually active men who did not accept a radical surgery. A conservative polichemotherapy treatment by ciclophosphamide, vincristine and prednisone has been proposed and performed for both cases and a complete resolution of disease was demonstrated. At the same time we assessed the erectile function by the IIEF score, before and after treatments.

## Introduction

The genital localization of lymphomas is actually a rare disease. We observed two patients affected by Non-Hodgkin Lymphoma (NHL); we chose a conservative treatment in order to obtain both a good quality of life and good oncological outcomes. We analysed the results with a review of the literature.

## Case Report

**Figure 1:**
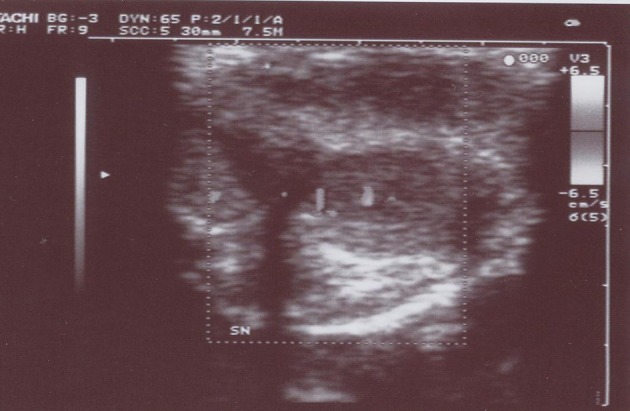
Ultrasonography of the lesion.

Over the last 12 months two men (71 years old and 72 years old, respectively) presented with a hard and elastic egg-shaped node of the penis. One of the patients had a history of several recurrences of NHL. We performed an ultrasonography (Fig. 1) with needle biopsy of the lesion in both patients [1] and the pathological examination showed in one case a NHL small cell type and in the other case a NHL mantellar cell type. An abdominal CT scan did not show any evidence of nodal involvement. The two patients were also assessed by the 15 questions-IIEF questionnaire which gave a score of 16 and 18, respectively. So a polichemotherapic treatment by ciclophosphamide, vincristine and prednisone has been decided. We repeated the therapy three times in 40 days. After that we reassessed the two patients by a physical examination and an ultrasonography of the penis. At that moment there was a complete resolution of penile NHL localisation in both patients, though in one of them a leucopenia and splenomegalia occurred and dissuaded from repeating other chemotherapic cycles.

After a follow-up of 4 and 8 months no local disease recurrences occurred.

Both patients did not complain any impairment of erectile function as we observed by the IIEF score which was still 15 in the first patient (16 before treatment) and 16 in the second one (16 before treatment).

## Discussion

Malignant lymphomas of the penis are rare [2-4] and not associated to specific symptoms or signs [5], even if they could present as small and egg-shaped node [6].

The disease is typical of adults [2], with just one paediatric case in the literature [7]. Surgical removal of these nodes is an easy and effective approach, but it frequently causes esthetical problems and erectile disfunction [8]. So our small experience demonstrated that a conservative treatment is effective both in terms of disease recurrence and functional organ preservation [6, 9].

For these reasons in penile lymphomas a conservative therapy could be considered as the first-choice approach also with a curative goal [10].
